# Supplementary material for: Climate and Land-Cover Change Impacts and Extinction Risk Assessment of Rare and Threatened Endemic Taxa of Chelmos-Vouraikos National Park (Peloponnese, Greece)
Source: Plants (Basel). 2022 Dec 15;11(24):3548. doi: 10.3390/plants11243548 (PMC9784511; doi:10.3390/plants11243548)
Supplement: Supplementary file 1 [file plants-11-03548-s001.zip › Figures Legends -Supplementary.pdf]

Figure S1. Land cover types and their anticipated transition between 2015 and 2100 in five-year intervals in the study area under the SSP5 RCP 85 LULC scenario. The far-left column refers to 2015 and the far-right column to 2100. Each bar shows the area each land cover type occupies in each year, while the flows between each five-year interval depict the anticipated transitions between the land cover types. BLDT: broad-leaf deciduous trees. BLES: broad-leaf evergreen shrubs. C3: C3 grasslands. NLDET: needle-leaf evergreen trees.

Figure S2. Intensity of land type change over the entire time-period (2015-2100) at five-year intervals between C3 grasslands and need-leaf evergreen trees under the SSP5 RCP 85 LULC scenario. The right panel depicts the rate of annual change, with the dashed line denoting the mean change rate between the different land cover types. The left panel depicts the percentage of change during the corresponding time step.

Figure S3. Chord diagram for the entire time-period (2015-2100) under the SSP5 RCP 85 LULC scenario.
