# Supplementary figures and images for: Climate and Land-Cover Change Impacts and Extinction Risk Assessment of Rare and Threatened Endemic Taxa of Chelmos-Vouraikos National Park (Peloponnese, Greece)
Source: Plants (Basel). 2022 Dec 15;11(24):3548. doi: 10.3390/plants11243548 (PMC9784511; doi:10.3390/plants11243548)

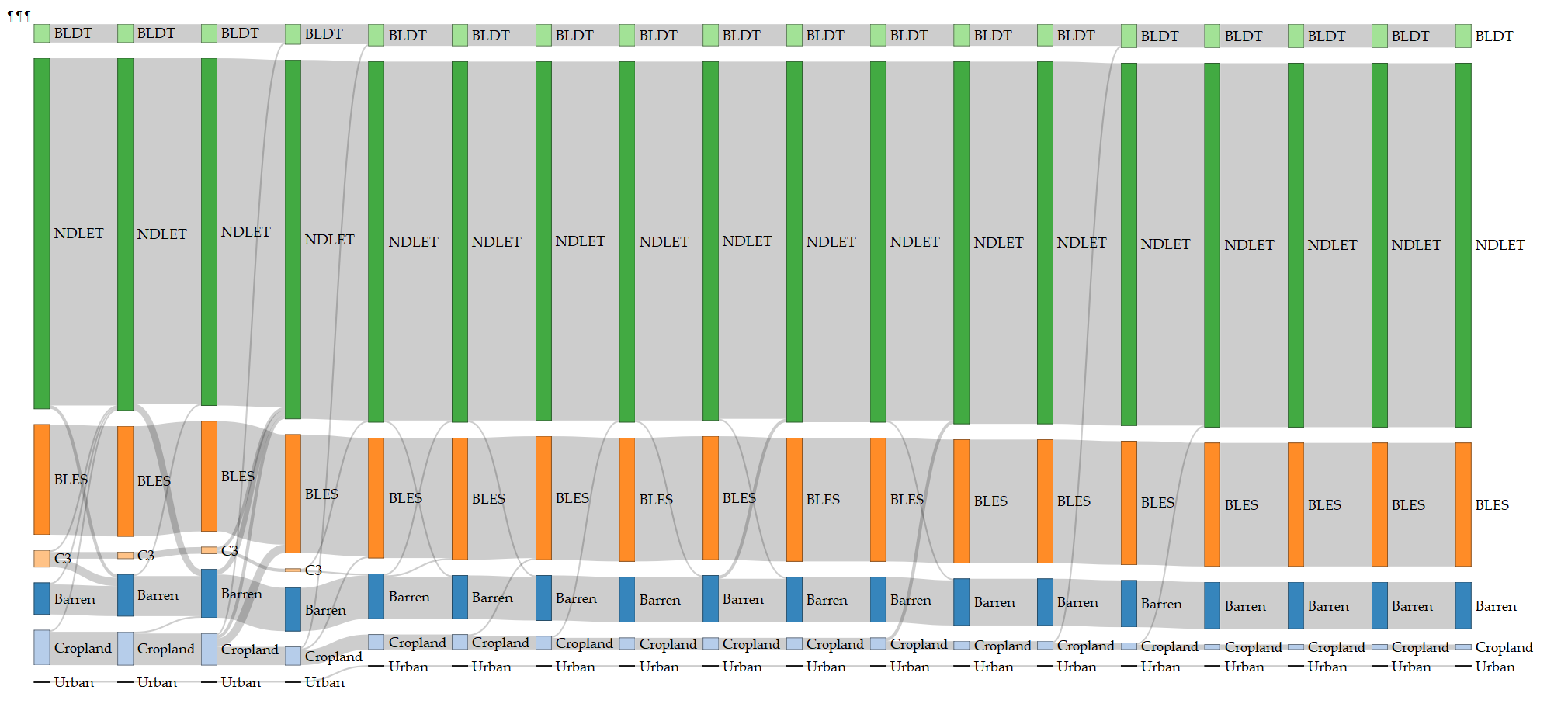

Supplement: Supplementary file 1 [file plants-11-03548-s001.zip › Figure S1.png]

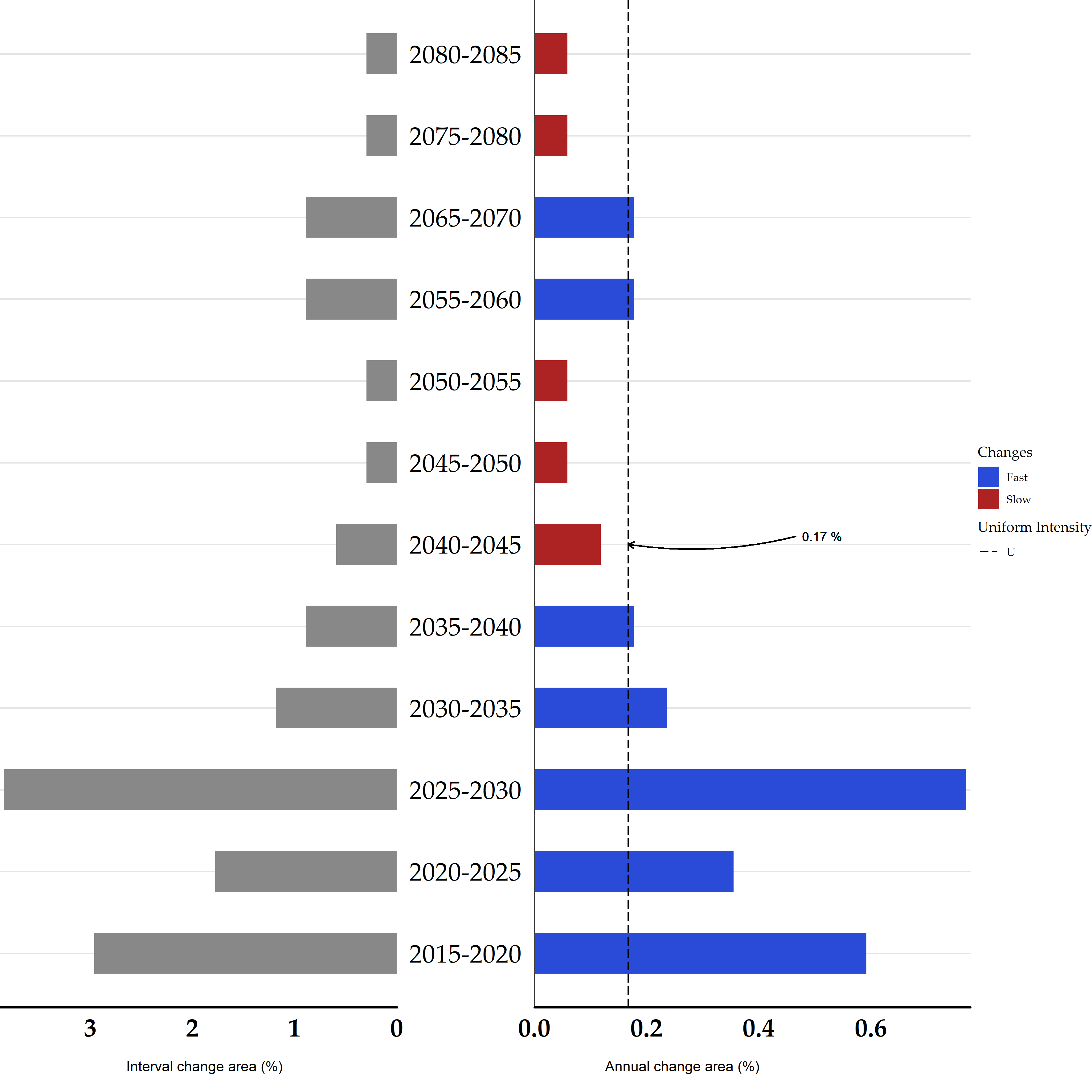

Supplement: Supplementary file 1 [file plants-11-03548-s001.zip › Figure S2.png]

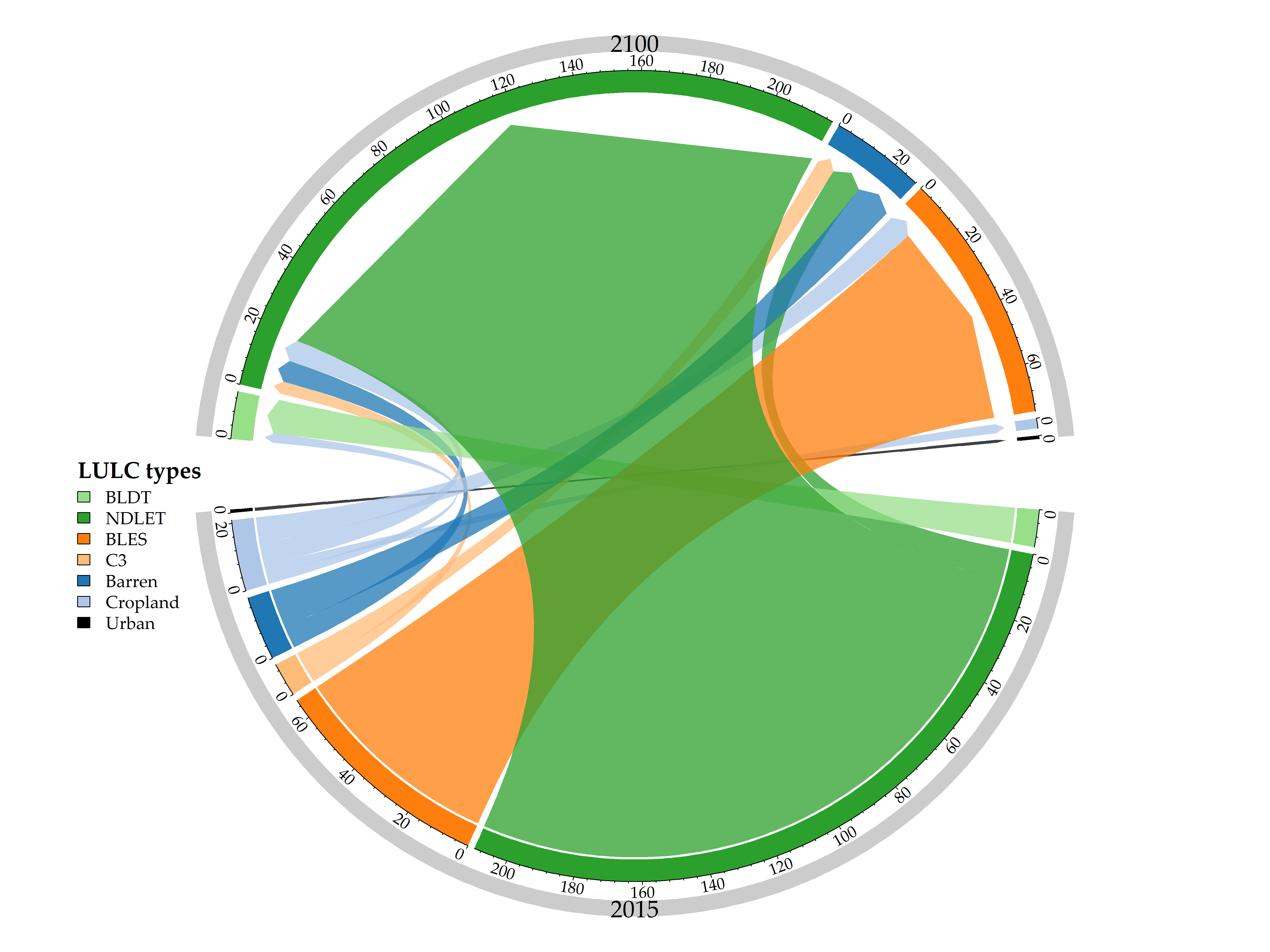

Supplement: Supplementary file 1 [file plants-11-03548-s001.zip › Figure S3.png]
